# Temporal Archive of Atmospheric Microplastic Deposition Presented in Ombrotrophic Peat

**DOI:** 10.1021/acs.estlett.1c00697

**Published:** 2021-10-25

**Authors:** D. Allen, S. Allen, G. Le Roux, A. Simonneau, D. Galop, V. R. Phoenix

**Affiliations:** † Department of Civil and Environmental Engineering, University of Strathclyde, Glasgow G11XJ, Scotland; ‡ Laboratoire écologie fonctionnelle et environnement, Université de Toulouse, CNRS, Toulouse 31062, France; § School of Geography, Earth and Environmental Sciences, University of Birmingham, Birmingham B15 2TT, England; ∥ Department of Earth and Environmental Sciences, Dalhousie University, Halifax, NS B3H 4R2, Canada; ⊥ ISTO, Université d’Orléans, CNRS UMR 7327, BRGM, 45100 Orléans, France; # GEODE, Université Toulouse Jean Jaurès, UMR-CNRS 5602, Toulouse 31062, France; ∇ LabEx DRIIHM, OHM Pyrénées Haut Vicdessos, ANR-11-LABX-0010, INEE-CNRS, Paris 75000, France

## Abstract

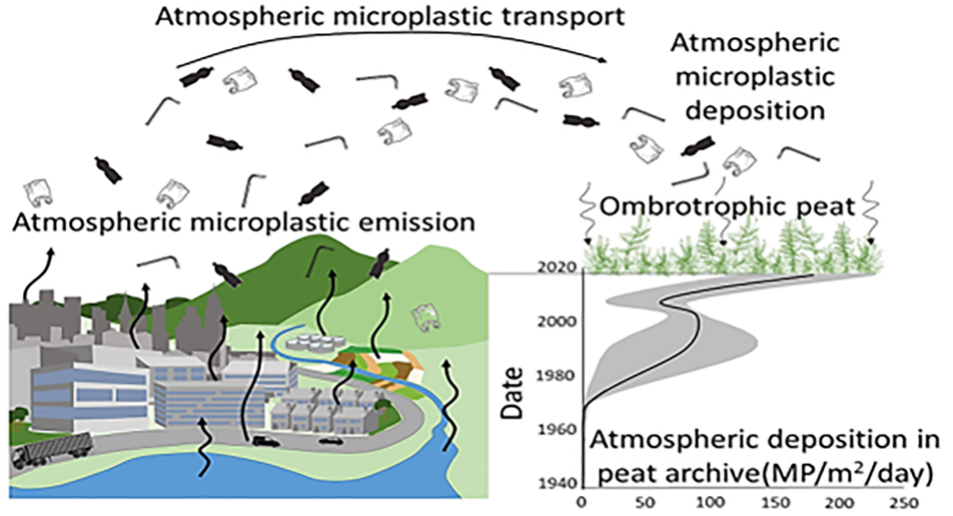

Ombrotrophic peatland—fed solely from atmospheric deposition of nutrients and precipitation—provide unique archives of atmospheric pollution and have been used to illustrate trends and changes in atmospheric trace element composition from the recent decadal to the Holocene period. With the acknowledgment of atmosphere plastic pollution, analysis of ombrotrophic peat presents an opportunity to characterize the historical atmospheric microplastic pollution prevalence. Ombrotrophic peatland is often located in comparatively pristine mountainous and boreal areas, acting as sentinels of environmental change. In this paired site study, a *Sphagnum* ombrotrophic peat record is used for the first time to identify the trend of atmospheric microplastic pollution. This high altitude, remote location ombrotrophic peat archive pilot study identifies microplastic presence in the atmospheric pollution record, increasing from <5(±1) particles/m^2^/day in the 1960s to 178(±72) particles/m^2^/day in 2015–2020 in a trend similar to the European plastic production and waste management. Compared to this catchment’s lake sediment archive, the ombrotrophic peat core appears to be effective in collecting and representing atmospheric microplastic deposition in this remote catchment, collecting microplastic particles that are predominantly ≤20 μm. This study suggests that peat records may be a useful tool in assessing the past quantities and trends of atmospheric microplastic.

## Introduction

Microplastic (MP) particles are 5 mm–1 μm “synthetic solid particle[s] or polymer matri[cies]”.^1,2^ Since the creation of Bakelite (1907), commercialization of PVC (1930s), and use of nylon fibers (1938)^1,3,4^ plastic creation, waste and mismanagement has increased relentlessly. In 2019, 368 million metric tonnes (Mt) was produced globally,^5^ with an estimated 32% of the municipal waste mismanaged and potentially lost to the environment (in 2016).^6^ This has resulted in a predicted 3-fold increase in plastic waste entering the environment by 2040 (∼80 Mt, business as usual scenario).^7,8^ This increase in plastic waste lost to the environment over past decades has been quantified in environmental archives of sediment (both freshwater lake and marine sediments), soil, and ice but has not previously been reported in peat.^9^

Marine sediments are the most commonly analyzed archives, with studies evidencing historical marine MP deposition in Arctic, Baltic, Mediterranean, North seas, Atlantic and Pacific ocean sediments.^10^ Mangrove sediments are areas of high marine deposition and have illustrated an exponential increase in MP deposition since the 1950s.^11,12^ Within urban lakes, similar exponential trends have been found, showing the most significant increase in plastic deposition to occur in the most recent decades (1980–present).^13,14^ Urban freshwater and marine sediment archives illustrate a notable amount of variation in MP quantities, both relative to the sample location and over the historic timeline.^10,13,15^ This suggests that marine and freshwater MP deposition is not consistent and may be influenced by availability, location, and environmental conditions.

Sea ice cores from both the Arctic and Antarctica illustrate historical MP to depths greater than 1 m.^16,17^ Sea ice collects MP from both the marine and atmospheric environments, often illustrating high uptake of MP from the surrounding seawater.^16−19^ Due to the remote location of some ice sample sites, it is suggested that atmospheric transport is a significant MP vector.^20^

Alternatively, assessment of the long-term plankton trawl records in the North Atlantic and adjacent seas illustrates that since the 1950s there has been a consistently increasing trend in marine plastic litter. The most significant increase was seen in the 1990s, with the greatest marine plastic litter recorded in plankton nets occurring between 2000 and 2009.^21^ These data sets all illustrate the increasing prevalence of MP in the environment (sea, sediment, soil), and it is logical to hypothesize a similar occurrence in an atmospheric archive.

Ombrotrophic peat has been used to illustrate changes in atmospheric composition and deposition of anthropogenic pollutants over the past centuries.^22,23^ Ombrotrophic peat collects and retains atmospheric dust, particles, and pollutants, providing dated historical records of past humans influence,^9,24,25^ such as mining, urbanization, and industrial activities (e.g., lead, fly ash, antimony, copper^26−29^), illustrating trends over the industrialization era.

Disaggregating purely atmospheric pollutants from other transport pathways (e.g., runoff, erosion, seawater, other (sub)surface influences) in sea ice, soil, or sediment records is difficult, and thus, defining solely the atmospheric influence is difficult. Ombrotrophic *Sphagnum* peatlands are unique as they receive pollutants, nutrients, and water solely from the atmosphere. Thus, despite inconsistent growth rates, bioturbation from plant roots, and potentially incomplete deposition retention,^9^ peat may provide a unique insight into the history of atmospheric MP pollution.

## Materials and Methods

The pilot study field location is a small remote catchment in the central Pyrenees. The Arbu catchment is 1.6 km^2^ (42°48′18″ N, 1°26′15″ E), at an elevation of 1940 m a.m.s.l (Figure S1). The site has low local hiking traffic, which does not traverse near the peatland, but it is acknowledged that local hikers may be a minor contributing factor to plastic in the catchment. The catchment has a lake at its base and an ombrotrophic peat area on the western elevation. A Wardenaar peat core (*N* = 1) was collected from the peat site during a field campaign in 2017, subsampled (∼1 cm deep sections) in the laboratory, and analyzed using μRaman spectroscopy following standard methods (detailed methods description in the SI). Ombrotrophic peat (hereafter “peat”) has not previously been used to quantitatively characterize historical MP trends, while lake archives are well established in identification of past trends in catchment MP pollution. To “logic check” the MP results found in the peat core, the results were compared with MP quantified in the Lake Arbu core (*N* = 1). The lake collects runoff and erosion from the total catchment as well as direct atmospheric MP deposition and is therefore more representative of the total catchment atmospheric MP deposition in contrast to the peat, which only represents deposition specifically onto the peat surface. The lake core was collected during a previous campaign (2014) using a UWITEC corer from a floating platform (subsampled core analysis following the same MP methods as implemented for peat analysis, detailed in the SI). Archive samples were dated using ^210^Pb and ^14^C radiocarbon dating techniques,^30−32^ and age depth models are created to date all subsamples (CLAM, CRS^33,34^) (SI).

μRaman analysis was undertaken using a Horiba Scientific Xplora Plus, using a 785 nm laser 50–3200 cm^–1^, 1.5 cm^–1^ resolution, 0.5 μm confocal imaging accuracy with an X–Y motorized stage.^35,36^ Approximately 30% of the filter surface was analyzed, collecting a minimum of 10 acquisitions of 15 s using a maximum of 25% power (filter) (1200 grating mm^–1^, 50 μm slit, modified as necessary to achieve effective spectra clarity). LOD/LOQ for this analysis was set to 5 μm. MP size and shape were characterized using Nile red fluorescence microscopy^37−39^ and FIJI software.

For both peat and lake samples, field blanks (negative controls) were created by randomly selecting subsamples from the bottom of the cores (dated pre-1900). Blank samples were processed following the (H_2_O_2_ organic digestion and ZnCl_2_ density separation, SI) peat/lake sample preparation protocol prior to filtration onto 0.2 μm pore, 25 mm diameter aluminum oxide filters. All sample preparation and analysis were completed in a controlled laboratory following rigorous protocols to mitigate contamination. Peat and lake samples were all blank corrected (SI).

## Results and Discussion

### Microplastic Particle Counts

**Figure 1:**
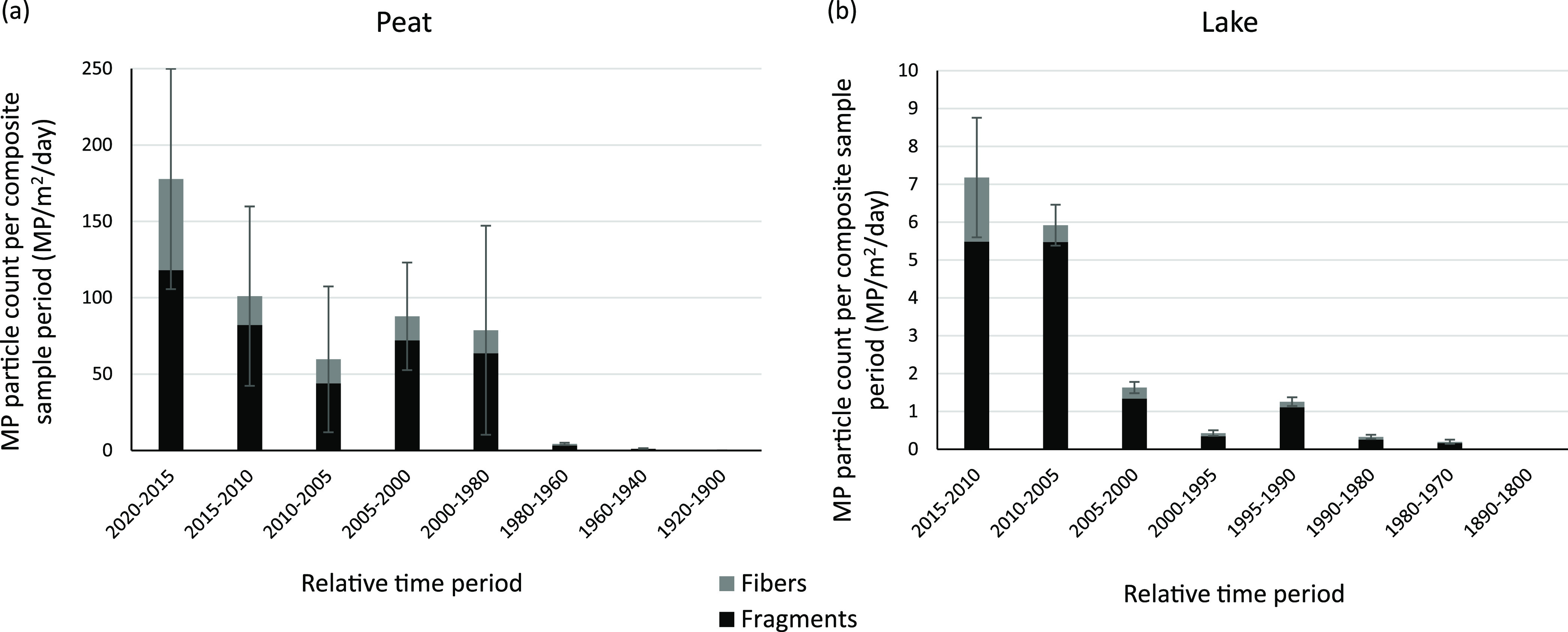
Microplastic content represented in the peat (a) and lake (b) cores collected from the Arbu Catchment. The MP quantities are represented as deposition rates^1^ for time periods relevant to collected subsamples of the cores and in units comparable to previously published environmental MP rates. All nonfibrous MP are classified as fragments. While peat samples were available to create 5-year time step analysis back to 2000 and 20-year time steps back to 1940, lake samples were constrained by sample volume availability for samples dated 1940–1970, and therefore, only the 1970–2015 samples were analyzed (alongside pre-1900 dates field process blanks). Error bars represent the standard deviation for the sample set. It is noted that the peat core results are actually net accumulation rates of MP illustrating the MP deposited and potentially lost through re-entrainment, but for the purposes of this paper, they are described as deposition.

The greatest quantity of MP was found in the top section of the single peat core, presenting a deposition rate of 178 (±79) MP/m^2^/day (Figure 1a). The quantity of MP decreased with depth, with negligible MP found in the 1940–1960 dated samples (1–2 MP/sample, ≤1 MP/m^2^/day). The change in the rate of MP deposition was greatest in the top sample (2015–2020) and 1980–2000 sample (deposition rate increase of >70 MP/m^2^/day). The proportion of MP fibers was greatest in the top peat sample (>30% of the total MPs), decreasing to ≤20% in the 1960–1980 samples. The decrease may be due directly to atmospheric concentration and deposition or in situ particle degradation (UV, chemical or mechanical forces) of particles caught in the peat (sub)surface, acknowledging the uncertainty due to taphonomic processes.^9^

Lake samples from the single lake core illustrated a similar decline in MP numbers with depth. The uppermost samples (2010–2015) present a deposition rate of 7.2 (±1.6) MP/m^2^/day (2620 (±577) MP/m^2^/year), equivalent to 2800 (±616) MP/kg (dry weight). These values are comparable to MP found in Arctic (≤6695 MP/kg),^40^ Tibetan Plateau (8–563 MP/m^2^),^41^ and remote Swiss mountain lake sediments (100–1300 MP/m^2^)^42^ and lower than published urban lake sediment MP findings (Tables S1 and S2). This declines to a deposition rate of less than 600 MP/m^2^/year prior to 2005 (equivalent to ≤900 MP/kg), with lower MP found in samples dated pre-1990s (<140 MP/m^2^/year, <300 MP/kg). The proportion of fiber MP was relatively consistent throughout the lake samples (8%–24%). The greater comparative peat surface fiber counts may be a result of effective vegetation capture of fibers (and particles) by peat and the potential for fibers to move more slowly or get detained in the catchment vegetation and soils prior to runoff conveyance to the lake. The overall difference between peat and lake MP counts may also be due to the evolution of vegetation cover (1990–2020 increased shrub and heathland, increasing the filtration role by vegetation). MPs may also float on the lake surface, discharging downstream, resulting in only a proportion of catchment MP deposited in lake sediment.

### Microplastic Particle Size Distribution

**Figure 2:**
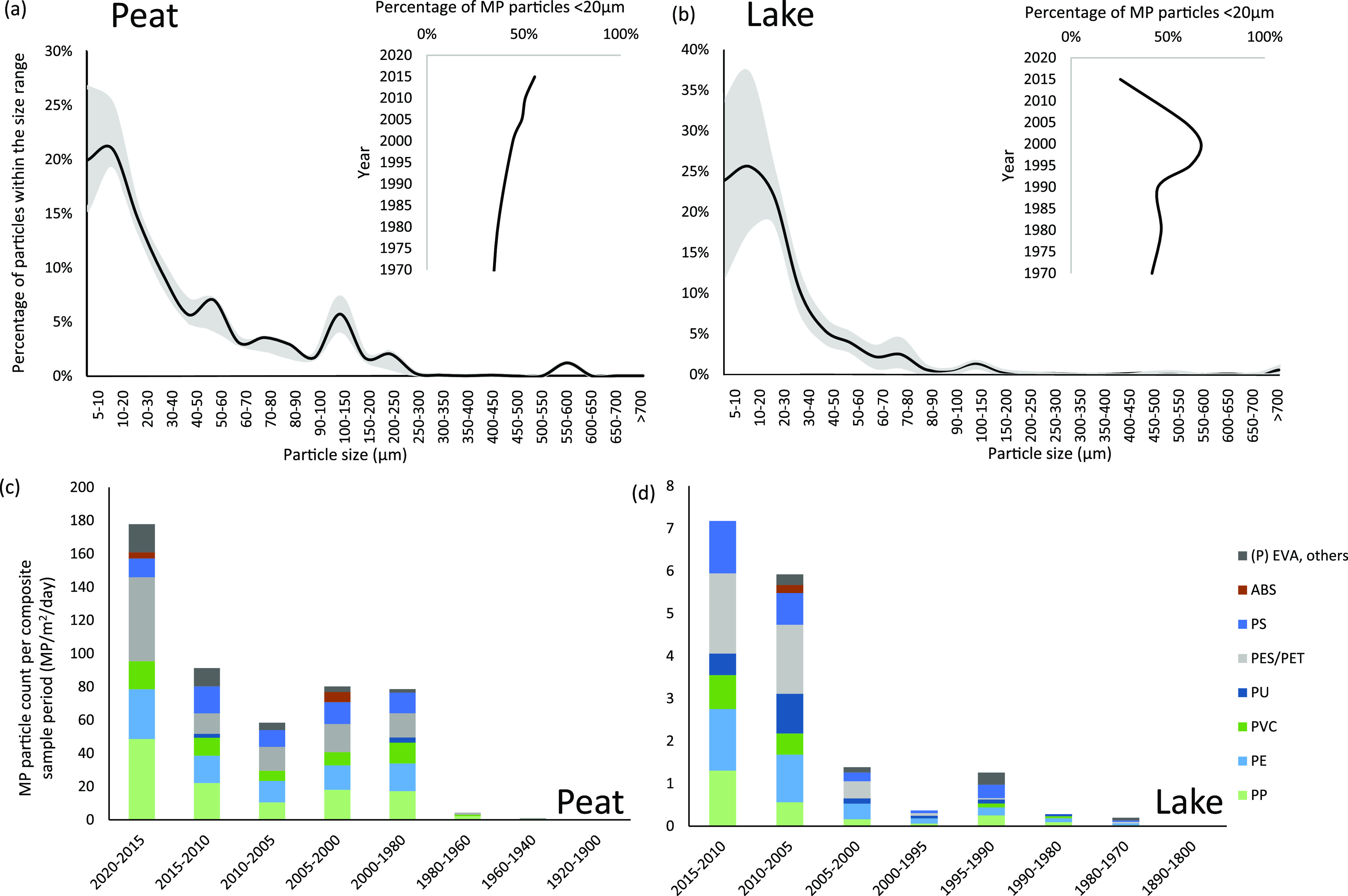
Microplastic particle size distribution for peat and lake samples (Figure 2a, b). The black line represents the mean particle size within the overall sample, and the gray shading is representative of the first–third quartile range of particle sizes. The particle size distribution results are disaggregated into fibers and fragments in Figures S3 and S5. The insets illustrate the proportion of smaller particles (MPs <20 μm) throughout the samples relative to the sample age dating. Polymer composition of samples, relative to the age depth are in Figure 2c and d and Figure S4).

Peat MP size distribution shows greater than 50% of particles identified as ≤30 μm (Figure 2). This concurs with the atmospheric deposition MP size distribution from direct atmospheric sampling for the adjacent catchment in the Pyrenees^36^ (>50% MP particles <20 μm). The overall prevalence of ≤30 μm MP suggests atmospheric deposition to primarily be smaller MP particles, reflective of the remote location. The proportion of MP less than 20 μm shows a slightly increasing trend since 1980 (SI), potentially due to an increase in creation/emission of smaller MP directly into the atmosphere from human activities (e.g., laundry (dryer) emissions, ineffective incineration, agricultural practices^43−45^) or/and an increase in macroplastic waste loss to the environment that has degraded over time (in situ or in transport) resulting in increased MP and an increase in atmospheric transport.

MP fragments (nonfibrous particles) follow the overall sample size distribution (Figure 2, Figures S3 and S5) with pre-1980 samples showing a greater proportion of larger fragments (>20 μm) than recent (top) samples (MP fragments less than 20 μm decline from 50% to 35% during 2020–1980) (SI). Microplastic fibers are generally between 20 and 200 μm in length with a predominant fiber length of 100–150 μm (Figure S3).

The lake core MP samples present a similar particle size distribution trend to peat. The proportion of less than 20 μm MPs shows little variation prior to 1995, an increase in smaller (<20 μm) particles post 1995, but a decrease in the top sample (2010–2015). This water–sediment interface difference may be a pre- and postburial difference. It may alternatively be due to a decrease in catchment influx of the smaller MP particles in surface runoff during this period (influenced by catchment vegetation change, increased shrub and heathland potentially detaining surface runoff MP), precipitation (e.g., fewer, less intense or shorter duration runoff events), and other meteorological conditions (e.g., lower average wind speed or planetary boundary layer).

### Plastic Types

**Figure 3:**
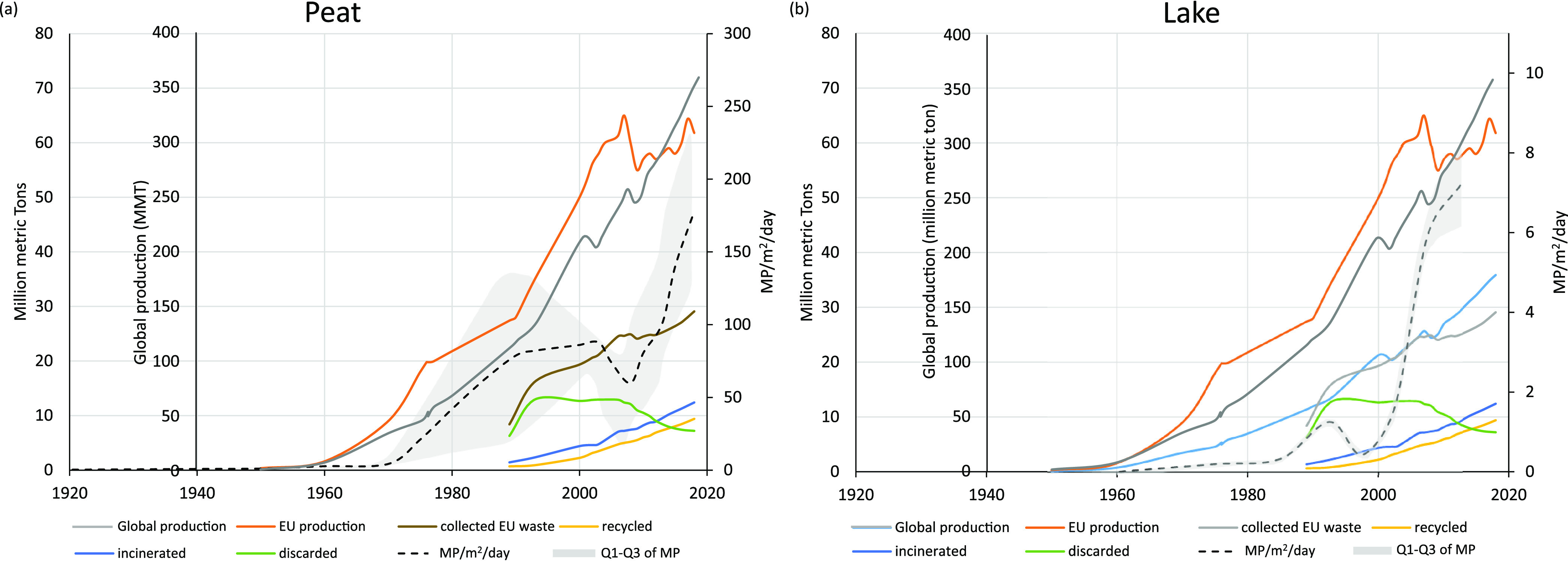
Comparison of peat and lake archive results with Europe and management trends. The global plastic production trend has been provided for supporting context to the European production trend. The EU and global trend data are compiled from published plastic statistics.^7,47^ Global production is indicated in gray, while European (EU) production is indicated in orange. EU collected waste is identified in brown, discarded (landfill) waste in green, incinerated waste in blue, and recycled plastic waste in yellow. The peat and lake archive results are presented as black dotted lines (average MP/m^2^/day) with the first–third quartile range shaded in light gray.

Polypropylene (PP), polyethylene terephthalate (PET), polyethylene (PE), polystyrene (PS), and polyvinyl chloride(PVC) are the most abundant in the peat core samples (Figure 3), generally following the European demand relative to polymer type.^7^ PE, PET, PP, and PVC show a general decreasing trend with depth, illustrating a relatively consistent increase in the atmospheric deposition of these polymers over time consistent with the increasing production and use of plastic. The sample MPs are composed of a complex mixture of polymer types, with ethylene vinyl acetate (EVA), polycarbonate (PC), acrylonitrile butadiene styrene (ABS), and other plastics occurring in the post 1980 samples. This concurs with the post 1980s development and commercialization of high performance plastics and the commercialization of low cost plastic personal devices and homecare products.^46^

The lake samples follow a similar decreasing trend in individual polymer prevalence and sample complexity with depth to that found in the peat archive. PS and PP indicate a localized increase in prevalence during 1990–1995, while PE and PET show a consistently declining trend with depth. In general, the peat and lake archives illustrate an increasing quantity of all plastic polymer types over time and a complexity in their composition.

### Past Trends of Atmospheric MP Deposition

Overall, the findings suggest peat to be an effective atmospheric MP deposition collector, illustrating a MP deposition rate (MP/m^2^/day) at a moderate interval (∼5 to 10 year time steps). Similar to lake archives, peat representation will fluctuate in resolution according to the availability of precipitation, growth nutrients, and climatic and environmental conditions. Peat growth and atmospheric MP deposition retention appears to be great enough to support atmospheric archive analysis of recent historic MP. Unlike passive or active field atmospheric sampling (e.g., collection via deposition collectors or pumped filters), it is acknowledged that an unknown proportion of atmospheric deposition may be resuspended, and taphonomic processes may influence the accumulation record,^9^ resulting in an under/overestimation of atmospheric deposition. This study confirms the effectiveness of ombrotrophic peat as a MP atmospheric deposition archive providing a unique record of past atmospheric MP deposition.

Acknowledging this single site pilot study, a comparative EU plastic trend assessment is used to tentatively expand on MP trends. Lake MP show a rapid MP increase post 2000, following plastic production trends. While MP generally increased from 1960 onward, the late 1990s MP lake deposition dip appears to coincide with plateauing of the discarded plastic waste stream (rather than sediment accumulation rate) and commencement of increased recycling and incineration/energy recovery waste processes.

Peat MP results show a similar increasing MP trend and a MP deposition dip between 2005 and 2010 coinciding with the recession (2007–2009) (less disposable income potentially resulting in lower single use plastic waste). This dip also coincides with the increase in EU collected waste plastic (∼3%) compared to the generally consistent increase in waste plastic collection and the drop in EU production. The MP dip in the 2005–2010 period also occurs alongside the start of the decrease in landfill (discarded) plastic waste in Europe (occurring from ∼2006 onward to the present). Interestingly, this dip is not seen in the lake archive, and this may be due to the lake MP being predominantly resultant from atmospheric deposition on the catchment which is then transported to the lake via surface runoff over vegetation and soil. The lag between atmospheric deposition and lake MP settling may result in a decrease in lake archive sensitivity (relative to time resolution), resulting in a smoother trend in the lake sediment archive (at this location and with this lake sediment deposition rate) compared to the atmospheric peat archive. The comparable peat MP, EU production, and waste trends tentatively suggest atmospheric MP concentration responds relatively quickly to changes in atmospheric MP emissions (primary and secondary) even where there is no significant local source (e.g., remote mountain sites). These comparisons of MP and EU plastic trends maybe an overinterpretation and require further investigation, but if atmospheric MP concentrations are found to respond quickly to decreased MP emissions, then management methods to help decrease atmospheric MP emissions could have a relatively immediate beneficial impact on MP pollution of the atmosphere and environment.

Both the pilot study and single core archive records of MP show some correspondence with European waste management, specifically the landfill/discarded plastic waste management that was predominant in the early plastic pollution era (pre-1995), the overall economic health (recession periods), and the quantity of plastic produced (especially in the EU), with a stronger comparison seen in the peat samples than the lake samples. While this is a pilot study, early results suggest MP peat monitoring may be sensitive enough to indicate atmospheric MP response to policy initiatives or global increases in certain polymer use. Further ombrotrophic peat archive analysis is needed for a variety of locations (urban to remote) to consolidate these results and provide a spatially comprehensive history of atmospheric MP composition and deposition.

## Supporting Information Available

Figure S1: contextualized site map, detailed methodology, commentary on peat, and a comparative sediment MP particle count with current published research. Tables (S1 and S2) of published sediment MP particle counts per mass of sediment and per m^2^ of sampled surface area. Figure S2: age date model information for Arbu Lake and peat cores and further graphical representation of MP particle size distribution (disaggregated for fibers and fragments), polymer types, MP particle size relative to age dated samples, and lake MP counts relative to MP/m^2^/year (Figures S3−S6) (PDF)
Additional detailed information (XLSX)
