# Supplementary material for: Temporal Archive of Atmospheric Microplastic Deposition Presented in Ombrotrophic Peat
Source: Environ Sci Technol Lett. 2021 Oct 25;8(11):954–60. doi: 10.1021/acs.estlett.1c00697 (PMC8582260; doi:10.1021/acs.estlett.1c00697)
Supplement: Supplementary file 1 — ez1c00697_si_001.pdf [file ez1c00697_si_001.pdf]

Temporal archive of atmospheric microplastic deposition presented in ombrotrophic peat

Allen, D., Allen, S., Le Roux, G., Simonneau, A., Galop, D. & Phoenix, V.R.

Supplementary information

Figure S1. Map and images of the field site location

Detailed methodology

Commentary on peat

Comparative sediment MP particle counts from published research

Table S1. Published sediment MP particle counts per mass of sediment sampled

Table S2. Published sediment MP particle counts per m^2 of sample surface area

Figure S2. Age date model information for the Peat and Lake cores collected from the Arbu Catchment

Figure S3. Microplastic particle size distributions for the Peat and Lake samples for fibres and fragments

Figure S4. Microplastic polymer types in the peat and lake archive samples

Figure S5. Microplastic particle size distribution relative to age dated samples

Figure S6. Lake MP counts relative to $MP/m^2/year$ to support direct comparison with previously published research

Figure S1. Map and images of the Field Site Location

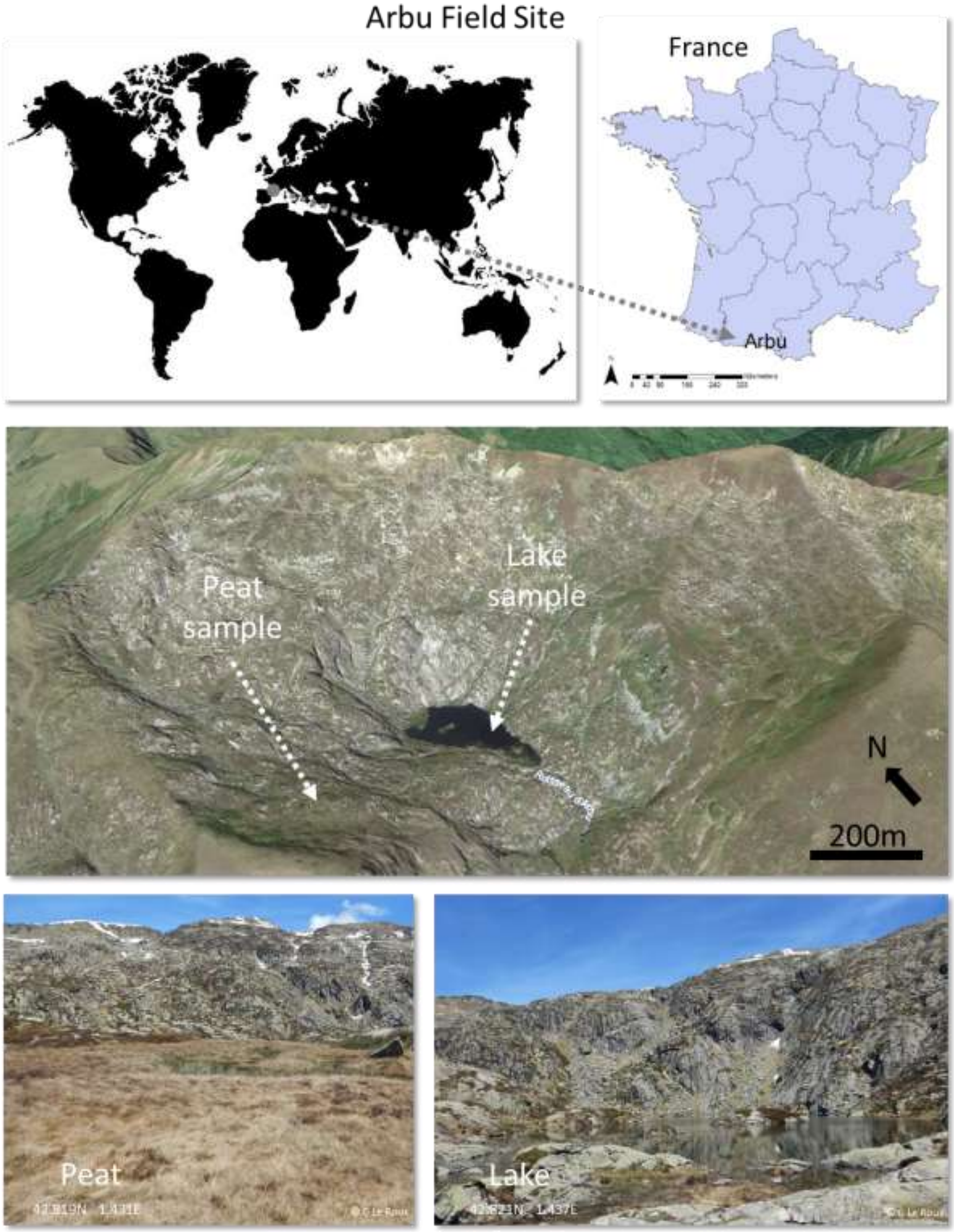

* Centre image, base capture: Google Earth V 9.142.0.1, Arbu Catchment, France, 42˚49’30”N, 1 ˚26’15”E, elevation 1,942m above mean sea level 3D map, viewed August 11, 2021, http://www.earth.google.com

Detailed Methodology

The pilot study field location is a small remote catchment in the central Pyrenees. The Arbu catchment is 1.6km^2 (42°48'18" N, 1°26'15" E), located adjacent to the Pic de Trois Seigneurs at an elevation of 1940m a.m.s.l. Ombrotrophic peat cores were collected from the field site during 2017 using a Wardenaar corer and following best practice sampling protocols[1]. Cores were wrapped and transported to the laboratory where they were frozen and then sub-sampled (~1cm sections) and stored in the freezer until analysis. During subsampling the outer section of the core was removed using metal or ceramic knives to minimize contamination. Sub-samples were then partitioned for analysis of ^{210}Pb and ^{14}C dating, elemental analysis, and analysis for microplastics.

Peat is a strong organic material, requiring extended sample preparation prior to spectroscopic polymer analysis. Approximately 5cm^3 samples (1cm in depth) were air dried for 48 hours (30˚C, foil wrapped to minimise contamination). Samples were not created by weight (g) but by surface area. Samples (in triplicate) were placed in borosilicate glass digestion vessels with glass caps (kiln sterilised at 300˚C for minimum 3hrs) placed in a heat block maintained at 50˚C. The organic material was digested using 10ml of hydrogen peroxide (30% w/w)[2–4] applied every 5 days until organic material had reduced sufficiently (no significant leaf or stem matter visible under 10x microscope on a 0.2µm 25mm Anodisc filter) (~15 days). Samples were then filtered onto 0.2µm pore 25mm diameter aluminium oxide (Anodisc) filters using kiln sterilized glass vacuum filtration. Glass digestion vessels and filtration funnels were rinsed 3 times with 250ml of MilliQ (18Ωcm) water to ensure removal of all sample material onto the filter. All the above steps occurred within a controlled laboratory environment and within a laminar flow vacuum hood pre-cleaned with bench surface covered in kiln sterilised aluminium foil, with all participants wearing 100% cotton laboratory coats and non-plastic clothing. Nitrile gloves were worn for handling of all chemicals, and all surfaces were cleaned with MilliQ water and covered with sterilised foil, changed between samples. All tools were kiln sterilised metal (no plastic tools used). All liquids used in the preparation process were double filtered through aluminium oxide filters to minimise potential contamination.

For comparison, a previously acquired lake core from Lake Arbu was analysed for microplastic abundance. The lake core was collected from the central most section of the lake. The core was recovered from beneath the lake floor using a UWITEC coring device operated from a floating platform [5–8]. Similar to the peat core, the lake core was sub-sectioned into ~1cm sections. These samples (10g ±1g) underwent the same organic material removal process described above. After organic digestion, the samples were filtered onto 0.45 µm polytetrafluoroethylene 47 mm diameter filter membrane with the digestion vessel rinsed 3 times with 250ml of MilliQ (18Ωcm) water. Samples were then placed in borosilicate glass density separation vessels (~10g/sample dry weight) with $ZnCl_2$ (1.6 g/ml density, pre-filtered through a 0.45 µm polytetrafluoroethylene membrane). Samples, in the density separation vessels, were mildly agitated for 7 days (60 revolutions/min, Edmund Buhler KS-15 shaker) (all caps, taps and outlets of the density separation vessels were wrapped in sterilised foil to limit atmospheric contamination during density separation). After 7 days, the settled material was drained from the density separation vessels and the remaining liquid+microplastic was filtered onto a 0.2µm pore 25mm diameter Anodisc (with the vessel flushed 3 times with 250ml of MilliQ (18Ωcm) water).

Cores were age-dated using ^{210}Pb and ^{14}C radiocarbon dating techniques[7,9,10]. ^{14}C dates were analysed for a base, mid and upper sample of each core by accelerator mass spectrometry and ^{210}Pb was analysed for subsamples (gamma spectrometry)[6]. Age depth models (CLAM, CRS[11,12]) were created using the ^{210}Pb and ^{14}C results and used to interpolate dates for each sample depth (Figure S2).

For both the peat and lake cores, several base samples (randomly selected from the lower section of the cores indicative of pre-1900) were analysed as full procedural field blanks (negative controls) as they were collected and analysed following the full analytical process. These ‘base’ core samples are all dated significantly before 1900 and therefore any plastic particles found in these samples are considered, for this study, entirely due to procedural contamination. Laboratory blanks (x3) were also created as full process blanks (using 250ml of MilliQ water as the ‘blank’ sample). Peat blanks presented 4MP/filter (±2.5), lake blanks presented 3.4MP/filter (±1.3) (less than 15% of the top sample plastic counts for all sample repetitions). Blank results were subtracted from all peat and lake final counts (blank correction).

All microplastic samples (peat and lake samples prepared and filtered onto 0.2µm pore Anodisc filters) were analysed to quantify the microplastic content using µRaman (Horiba Scientific Xplora Plus, 50-3,200cm^{-1}, 785nm laser, 1.5cm^{-1} resolution, 0.5µm confocal imaging accuracy with X-Y motorised stage)[4,13]. Samples were analysed following cross sections of both axes sampling format defined in Huppertsberg and Knepper (2018)[14], resulting in approximately 30% of the filter surface. It was assumed that the sampled 30% filter area was representative of the total sample with regards to homogeneity (due to a variety of centre location, filter edge and the area in between being sampled in the cross section sampling format of Huppertsberg and Knepper (2018)[14]). A direct, linear, extrapolation was made from the 30% filter area analysis result, following the equation: $x = y \times 3.33$, where y is the number of MP particles identified in the analysed 30% of the filter following the Huppertsberg and Knepper (2018) cross section sampling format, and x is the total extrapolated number of MP particles for the total filter. µRaman spectroscopic analysis used the 785nm laser (spatial resolution of 1µm) collecting a minimum of 10 acquisitions of 15s using a maximum of 25% power (filter) (1,200 grating mm-1, 50µm split, modified as necessary to achieve effective spectra clarity). LOD/LOQ for this analysis was set to 5µm. Individual µRaman spectra analysis was completed using open source Spectragryph software, the SLOPP and SLOPPE databases supplemented with an in-house new and environmentally degraded plastics library and multivariate analysis[15,16]. Plastic particle identification required a ≥80% spectral match. Only plastic polymer spectra were considered in this analysis, particles identified as containing synthetic dyes or similar were not included in the MP count unless there was also a ≥80% spectral match to a plastic polymer spectra. Due to the highly organic nature of peat and the digestion process necessary to separate microplastic the non-plastic particles (organic fragments) were not counted and included in this study. In future, research should include consideration of other non-plastic anthropogenic particles such as black carbon. µRaman analysis provided the particle counts for all samples and blanks. Sample filters were then stained with Nile Red (Cas No: 7385-67-3, Sigma-Aldrich, 1mg/L in acetone stock solution) and analysed by fluorescence microscopy following current best practice particle identification criteria (homogeneous, unnatural shape, no cellular structure, shiny/glassy, fluorescence above background threshold)[17–20]. Fluorescent images were analysed using ImageJ/FIJI software to identify the shape and size dimensions of the plastic particles on each filter (following the same cross sectional scanning method identified in in Huppertsberg and Knepper (2018)[14] and to provide a complementary plastic particle count check. The definition of a fibre in the study is particles with a length ratio of 1:3 or greater.

Commentary on peat

Recent studies have started to quantify the temporal change in atmospheric pollutants that form part of the ‘Anthropocene’ epoch[21–23] (for example polychlorinated biphenyl, a human created carcinogenic chemical found electrical and hydraulic equipment, pigments and dyes and used as a plasticiser in plastic[24,25]). Peat vegetation is noted to be effective in catching and collection

environmental MP[26,27] and to potentially act as an effective archive representation of historic atmospheric microplastic deposition[28]. As such, ombrotrophic peat archives may provide an effective medium to consider historic atmospheric microplastic content. Ombrotrophic peat can illustrate the historic atmospheric MP composition and concentration, illustrate the past trends in atmospheric MP, potentially highlight the influence of any policy or plastic use changes, and help forecast possible in atmospheric MP prevalence.

Physical, chemical and biological taphonomy processes are expected influence an ombrotrophic peat MP record, in a similar manner to their influence of sea ice, soil and sediment archive records of MP. Taphonomy is the processes affecting how MP are transported to, deposit and become 'permanently' detailed or stored within archive environments such as sediment, soil, ice and peat[28–30]. With regards to ombrotrophic peat, these processes include atmospheric transport of MP, dry deposition and precipitation onto a ombrotrophic peat surface, detention of MP particles within the peat vegetation and retention of these particles so that they form part of the archive as the peat continues to grow and decay. Within the detention and retention, processes that may influence the peat archive record include in-situ degradation due to the peat acidity (potentially a consideration for more acid sensitive MP such as Nylon 6), potentially inconsistent consolidation of surface (most recently deposited) MP and bioturbation due to root growth[28]. While the influence of taphonomy is demonstrated in some soil, sediment and ice archives, this has not been examined in any detail in peat archives yet and is an important area of future research. These processes may result in a bias, under or over representation of MP along the archive timeline or may help explain some of the trends or fluctuations potentially found in MP archive datasets.

<u>Comparative sediment MP particle counts from published research</u>

Table S1. Published sediment MP particle counts per mass of sediment sampled

**Location**	**Sample description**	**MP count per kg sediment**	**Limit of Quantification**	**Comparative value found in Lake Arbu***	**Reference**
Donghu Lake, China	Urban lake	≤ 7707 MP/kg	100µm	>163 MP/kg	[31]
Wuliangsuhai Lake China	Urban/agricultural lake	≤ 724 MP/kg	>500µm	~58 MP/kg	[32]
Lake Ontario, Canada	Urban lake	87-616 MP/kg	>500µm	~58 MP/kg	[33]
East China Sea	Coastal sediment core	≤ 7746 MP/kg	>50µm	~581 MP/kg	[34]
Three Gorges Dam, China	Dam sediment	25-300 MP/kg	<500µm	<82 MP/kg (LOQ of 200µm used)	[35]
Edgebaston Lake, UK	Urban lake	260 MP/kg	>500µm	~58 MP/kg	[36]
Lake Simcoe, Canada	Fishing and recreational lake	≤ 1070 MP/kg	>45µm	~581 MP/kg	[37]
Lake Sassolo, Switzerland	Remote mountain lake	547 MP/kg	>125µm	~163 MP/kg	[38]

Norwegian freshwater lakes	Semi-urbanised Lakes	7000 MP/kg	>36µm	~1242 MP/kg	39
London, England	Urban lake sediment	539 MP/kg	<500µm	<82 MP/kg (LOQ of 200µm used)	40
Lake Bolsena, Italy	Urban, agricultural and recreational lake	112 MP/kg	<300µm	<82 MP/kg (LOQ of 200µm used)	41
Lake Chiusi, Italy	Recreational and nature reserve lake	234 MP/kg	<300µm	<82 MP/kg (LOQ of 200µm used)	41
Taihu, China	Urban/agricultural lake	11-234 MP/kg	~100µm	~163 MP/kg	42
Poyang Lake, China	Urban, agricultural and recreational lake	54-506 MP/kg	<100µm	<163 MP/kg	43
Lake Ulansuhai, China	Urban, agricultural and recreational lake	14-24 MP/kg	<500µm	<58 MP/kg	44
Lake Onego, Russia	Urban lake sediment	2188 MP/kg	~100µm	~163 MP/kg	45
Rawal Lake, Pakistan	Urban lake sediment	700-1500 MP/kg	~200µm	<82 MP/kg	46
Ox-Bow Lake, Nigeria		347-7593 MP/kg	~20µm	<2087 MP/kg	47
Lake Mjøsa, Norway	Urban, agricultural and natural lake	10-1460 MP/kg	~75µm	~311 MP/kg	48
Arctic Sea	Sea sediment samples	≤ 6695 MP/kg	> 11µm	~2590 MP/kg	49

* Comparative Lake Arbu sediment values are calculated using a 'worst case' scenario, using the maximum percentage of microplastic particles found throughout the samples within the prescribed size range (limit of detection to 5mm) rather than the top sample percentage. The published lake and sediment studies listed here are not exhaustive but are recent studies with published limits of quantification.

There are limited remote area lake sediment studies published that considered sediment samples or archive sediment cores. Therefore, to provide context for the sediment sample results found within this remote mountain study (Lake Arbu) a range of published surface sediment (≤5cm top sediment sampled) are presented in Table S1. Given the wide range of particle size limitations within the published studies, the Lake Arbu sediment MP results have been calculated relative to the published study limits of quantification (Comparative value found in Lake Arbu column). It is evident that this study presents MP particle counts *per kg* that are in the lower range of published urban lake, Arctic sea and remote mountain lake sediment MP values. The Lake Arbu sediment MP content is lower than that found in the remote mountain Lake Sassolo (Switzerland) and the nature reserve Lake Chiusi (Italy). This provides some context to the sediment MP counts, suggesting this remote Pyrenees lake to contain lower MP particles per kg sediment compared to previously published lakes.

While many studies present results as MP particle count per kg of sediment sampled, many studies alternatively present MP particle count per area of sediment sampled. To enable future comparisons,

peat MP atmospheric deposition direct comparison and a more comprehensive contextualisation of the results, the Arbu study presents results as both MP/kg and MP/m^2.

Table S2. Published sediment MP particle counts per m^2 of sample surface area

Location	Sample description	MP count per m^2 sediment	Limit of Quantification	Comparative value found in Lake Arbu*	Reference
Vembanad Lake, India	Urban, agricultural lake sediment	96-496 MP/m^2	>200µm	<76 MP/m^2	[50]
Lake Huron, Canada	Urban, agricultural lake sediment	38 MP/m^2	<5mm	<51 MP/m^2 (0 MP/m^2 if LOQ is 1mm)	[51]
Lake Geneva, Switzerland	Urban influenced lake sediment	100-1300 MP/m^2	>300µm	<76 MP/m^2	[52]
Lake Garda, Italy	subalpine lake beach sediment	1108 ± 983 MP/m^2	~500µm (one subsample ~9µm)	~55 MP/m^2	[53]
Siling Co basin, Tibet plateau, China	Remote lake sediment	8-563 MP/m^2	<500µm	<544 MP/m^2 (LOQ of 50µm used)	[54]
Dutch continental shelf	Marine inshore sediment	99 ± 110 MP/m^2	~300µm	<76 MP/m^2	[55]
Belgian continental shelf	Marine inshore sediment	301 ± 445 MP/m^2	~245µm	<76 MP/m^2	[55]
English Channel	Marine inshore sediment	121 ± 144 MP/m^2	~260µm	<76 MP/m^2	[55]
French area of English Channel	Marine inshore sediment	131 ± 154 MP/m^2	~62µm	<544 MP/m^2	[55]
Swiss Lakes	Shoreline sediments	20-7200 MP/m^2	>300µm	<76 MP/m^2	[56]
Lake Bolsena, Italy	Lake shoreline sediment	1922± 662 MP/m^2	<300µm	<544 MP/m^2 (LOQ of 50µm used)	[41]
Lake Chiusi, Italy	Lake shoreline sediment	2117± 695 MP/m^2	<300µm	<544 MP/m^2 (LOQ of 50µm used)	[41]
Setúbal Lake, Argentina	Urban lake sediment	704 MP/m^2	~500µm	<76 MP/m^2	[57]
Arctic lake, Ny-Alesund, Svalbard	Arctic lakes sediment	~90 MP/m^2	<300µm	<76 MP/m^2 (LOQ of 200µm used)	[58]
Qinghai Lake, china	Remote lake	50-1292 MP/m^2	112µm	<153 MP/m^2	[59]
Red Hills Lake, India	Urban lake sediment	5-38 MP/m^2	~300µm	<76 MP/m^2	[60]

* Comparative Lake Arbu sediment values are calculated using a 'worst case' scenario, using the maximum percentage of microplastic particles found throughout the samples within the prescribed size range (limit of

detection to 5mm) rather than the top sample percentage. The published lake and sediment studies listed here are not exhaustive but are recent studies with published limits of quantification.

Lake Arbu sediment MP content is illustrated to generally fall at the lower extent of published lake sediment MP content *per area* (MP/m^2). Lake Arbu sediment MP content is comparable to the remote Siling Co basin lakes MP content in the Tibet plateau (China) and to the MP sediment content found in the Arctic lake in Svalbard.

<u>Figure S2. Age date model information for the Peat and Lake cores collected from the Arbu Catchment</u>

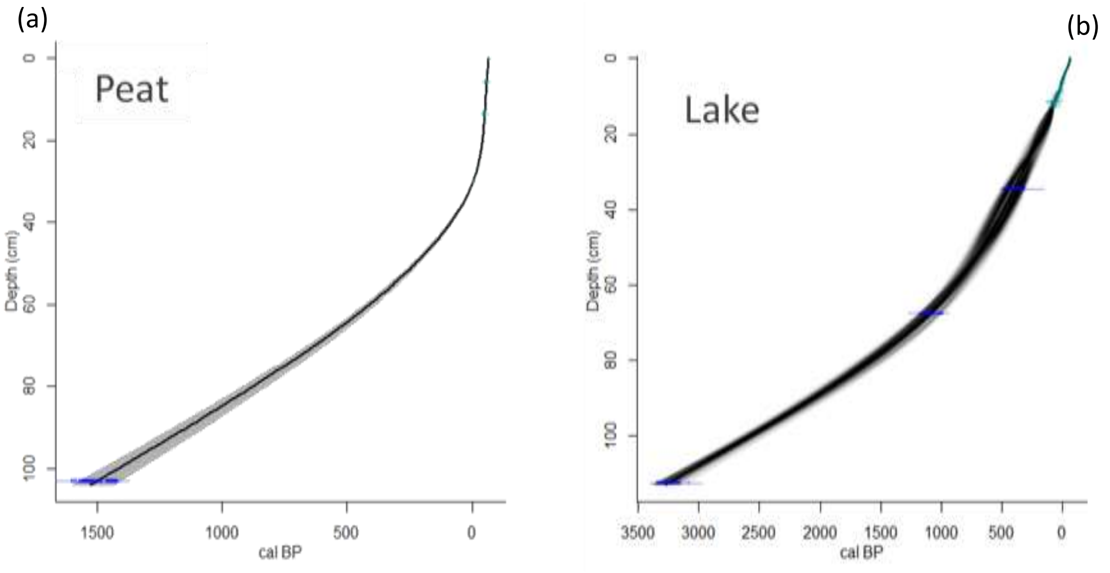

Figure S2. Age date model information for the Peat and Lake cores collected from the Arbu Catchment. S1(a) presents the modelled age date for the full depth of the peat core. Similarly, the modelled age depth for the lake core is presented in S1(b) (Marquer et al. 2020[9]). CLAM and CRS models were run to provide these results using ^{14}C and ^{210}Pb data respectively[61–63]. It is noted that there is no enrichment at the acrotelm/catotelm (the oxic/anoxic) boundary as is often found with other elements such as mercury. The acrotelm/catotelm boundary is found at approximately 45cm depth (in the Arbu peatland), below the 1950 depth of potential atmospheric MP deposition.

Figure S3. Microplastic particle size distributions for the Peat and Lake samples for fibres and fragments

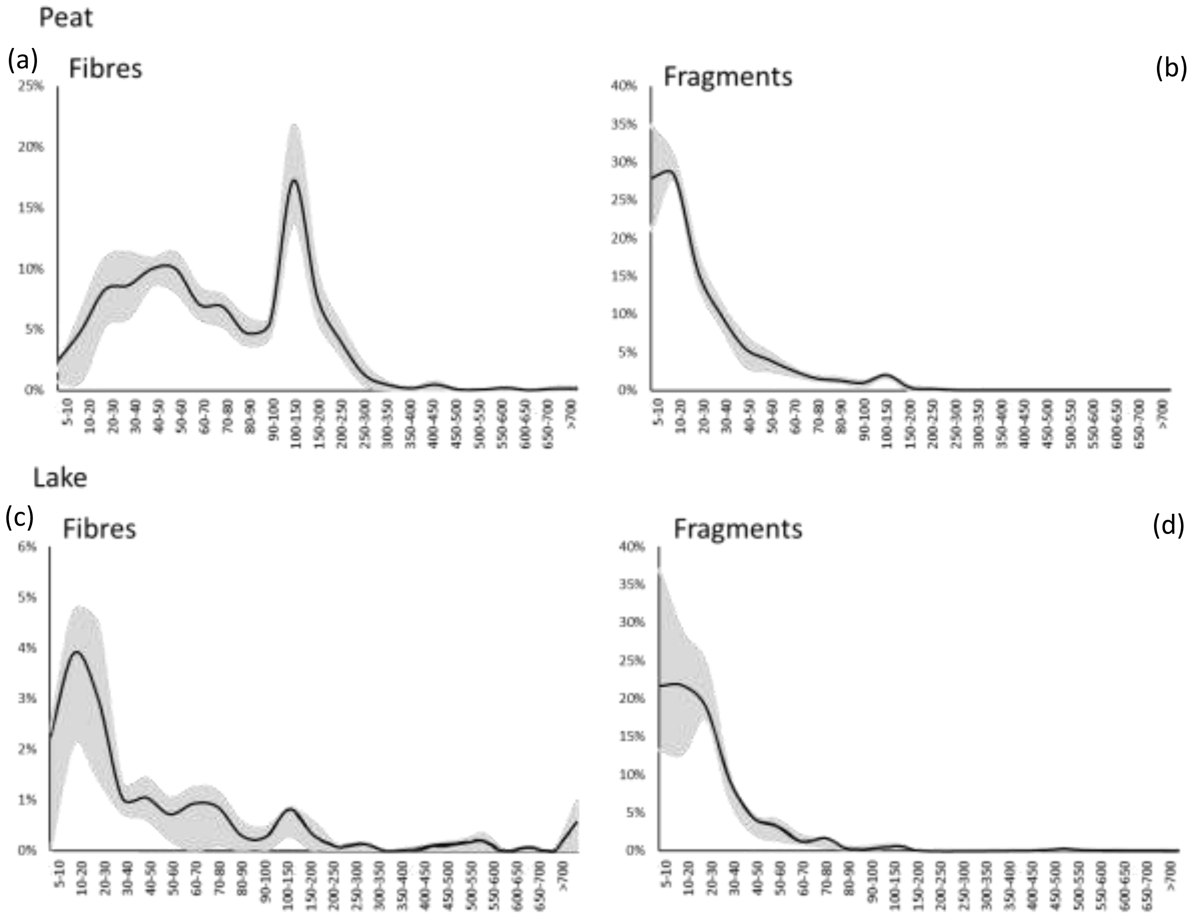

Figure S3. Microplastic particle size distributions for the Peat (S3 a and b) and Lake (S3 c and d) samples for fibres (a, c) and fragments (b, d) respectively. The grey shading illustrates the 1st to 3rd quartile range of particle size distribution; the black line represents the mean particle size distribution. Particle sizes are in diameter for the fragments and length for the fibres.

Figure S4. Microplastic polymer types in the peat and lake archive samples

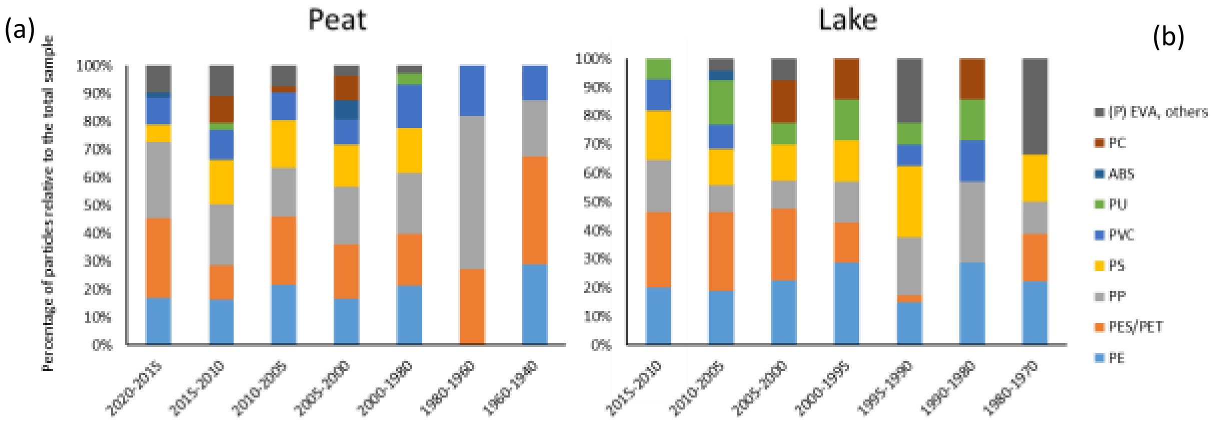

Figure S4. Microplastic polymer types in the peat (S4a) and lake (S4b) archive samples. The proportion of each polymer is represented for each of the samples with identified microplastic counts. Microplastics were identified using µRaman spectroscopy.

Figure S5. Microplastic particle size distribution relative to age dated samples

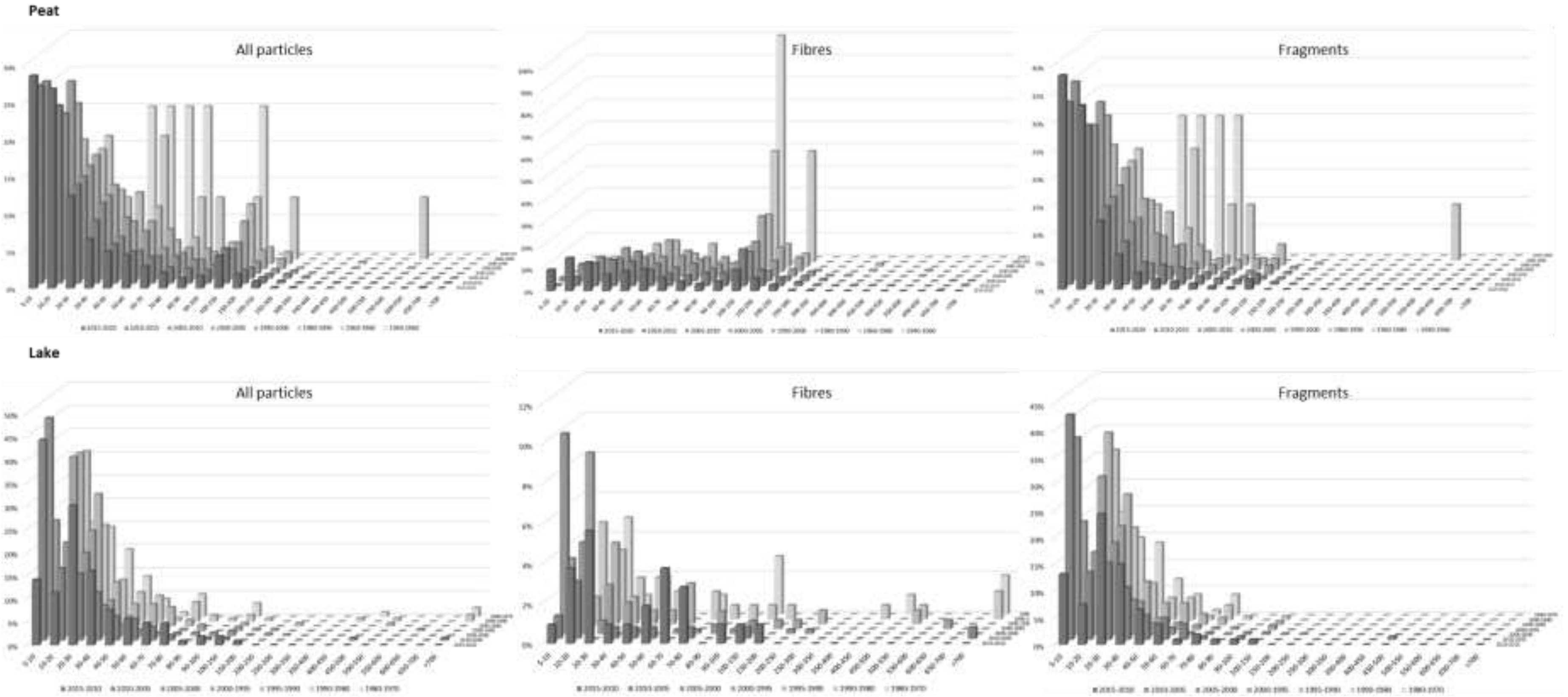

Figure S5. Microplastic particle size distribution for all particles, fibres and fragments relative to the age dated samples. The older dated samples (pre 1990 for lake samples and pre-1980 for peat samples) have fewer MP in the sample and therefore the size distribution is less dynamic .

Figure S6. Lake MP counts relative to MP/m2/year to support direct comparison with previously published research

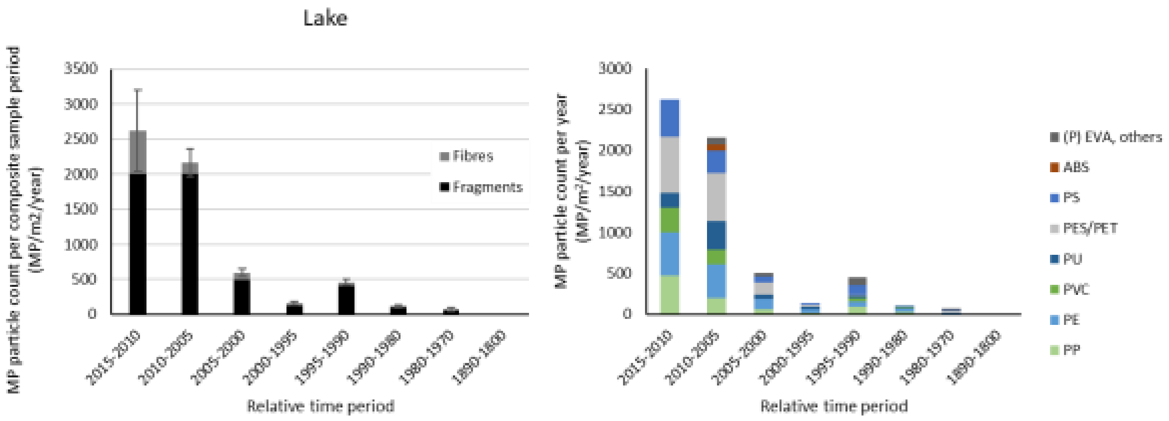

References

(1) De Vleeschouwer, F.; Chambers, F. M.; Swindles, G. T. Coring and Sub-Sampling of Peatlands for Palaeoenvironmental Research. *Mires Peat* **2010**, *7*, 621–628. https://doi.org/10.4028/www.scientific.net/MSF.508.621.

(2) Hurley, R.; Lusher, A. L.; Olsen, M.; Nizzetto, L. Validation of a Method for Extracting Microplastics from Complex, Organic-Rich, Environmental Matrices. *Environ. Sci. Technol.* **2018**, *52* (13), 7409–7417. https://doi.org/10.1021/acs.est.8b01517.

(3) Zhang, Y.; Kang, S.; Allen, S.; Allen, D.; Gao, T.; Sillanpaa, M. Atmospheric Microplastics: A Review on Current Status and Perspectives. *Earth-Science Rev.* **2020**, *203*, 103118. https://doi.org/10.1016/j.earscirev.2020.103118.

(4) Allen, S.; Allen, D.; Phoenix, V. R.; Le Roux, G.; Duranteza, P.; Simonneau, A.; Stéphane, B.; Galop, D. Atmospheric Transport and Deposition of Microplastics in a Remote Mountain Catchment. *Nat. Geosci.* **2019**, *12*, 339–344. https://doi.org/10.1038/s41561-019-0335-5.

(5) Arnaud, F.; Poulenard, J.; Giguet-covex, C.; Wilhelm, B.; Revillon, S.; Jenny, P.; M, R.; Enters, D.; Bajard, M.; Fouinat, L.; Doyen, E.; Simonneau, A.; Pignol, C.; Chapron, E.; Vanniere, B.; Sabatier, P. Erosion under Climate and Human Pressures : An Alpine Lake Sediment Perspective. *Quat. Sci. Rev.* **2016**, *152*, 1–18. https://doi.org/10.1016/j.quascirev.2016.09.018.

(6) Simonneau, A.; Chapron, E.; Courp, T.; Tachikawa, K.; Le Roux, G.; Baron, S.; Galop, D.; Garcia, M.; Giovanni, C. Di; Motellica-heino, M.; Mazier, F.; Foucher, A.; Houet, T.; Desmet, M.; Bard, E. Recent Climatic and Anthropogenic Imprints on Lacustrine Systems in the Pyrenean Mountains Inferred from Minerogenic and Organic Clastic Supply (Vicdessos Valley, Pyrenees, France). *The Holocene* **2013**, *23* (12), 1764–1777. https://doi.org/10.1177/0959683613505340.

(7) Doyen, E.; Bégeot, C.; Simonneau, A.; Millet, L.; Chapron, E.; Arnaud, F.; Vannière, B. Land Use Development and Environmental Responses since the Neolithic around Lake Paladru in the French Pre-Alps. *J. Archaeol. Sci. Reports* **2016**, *7*, 48–59. https://doi.org/10.1016/j.jasrep.2016.03.040.

(8) Allen, D.; Simonneau, A.; Le Roux, G.; Mazier, F.; Marquer, L.; Galop, D.; Binet, S. Considering Lacustrine Erosion Records and the De Ploey Erosion Model in an Examination of Mountain Catchment Erosion Susceptibility and Precipitation Reconstruction. *Catena* **2020**, *187* (September), 104278. https://doi.org/10.1016/j.catena.2019.104278.

(9) Marquer, L.; Mazier, F.; Sugita, S.; Galop, D.; Houet, T.; Faure, E.; Gaillard, M. J.; Haunold, S.; de Munnik, N.; Simonneau, A.; De Vleeschouwer, F.; Le Roux, G. Pollen-Based Reconstruction of Holocene Land-Cover in Mountain Regions: Evaluation of the Landscape Reconstruction Algorithm in the Vicdessos Valley, Northern Pyrenees, France. *Quat. Sci. Rev.* **2020**, *228*. https://doi.org/10.1016/j.quascirev.2019.106049.

(10) Simonneau, A.; Chapron, E.; Vanniere, B.; Wirth, S. B.; Gilli, A.; Di-giovanni, C.; Anselmetti, F. S.; Desmet, M.; Magny, M. Mass-Movement and Flood-Induced Deposits in Lake Ledro , Southern Alps , Italy : Implications for Holocene Palaeohydrology and Natural Hazards. *Clim. Past* **2013**, *9*, 825–840. https://doi.org/10.5194/cp-9-825-2013.

(11) Blaauw, M. Methods and Code for “classical” Age-Modelling of Radiocarbon Sequences. *Quat. Geochronol.* **2010**, *5* (5), 512–518. https://doi.org/10.1016/j.quageo.2010.01.002.

(12) Appleby, P. G. Dating of Sediments by 210Pb: Problems and Solutions. In *Proceedings of a*

seminar 'dating of sedilents and determination of sedimentation rate, STUK A145; Helsinki, 1997; pp 7–24.

(13) Wright, S. L.; Levermore, J. M.; Kelly, F. J. Raman Spectral Imaging for the Detection of Inhalable Microplastics in Ambient Particulate Matter Samples. *Environ. Sci. Technol.* **2019**. https://doi.org/10.1021/acs.est.8b06663.

(14) Huppertsberg, S.; Knepper, T. P. Instrumental Analysis of Microplastics—Benefits and Challenges. *Anal. Bioanal. Chem.* **2018**, *410* (25), 6343–6352. https://doi.org/10.1007/s00216-018-1210-8.

(15) Munno, K.; Frond, H. De; Donnell, B. O.; Rochman, C. M. Increasing the Accessibility for Characterizing Microplastics : Introducing New Application-Based and Spectral Libraries of Plastic Particles (SLoPP & SLoPP-E). *Anal. Chem.* **2020**, *92* (3), 2443–2451. https://doi.org/10.1021/acs.analchem.9b03626.

(16) Levermore, J. M.; Smith, T. E. L.; Kelly, F. J.; Wright, S. L. Detection of Microplastics in Ambient Particulate Matter Using Raman Spectral Imaging and Chemometric Analysis. **2020**. https://doi.org/10.1021/acs.analchem.9b05445.

(17) Shim, W. J.; Song, Y. K.; Hong, S. H.; Jang, M. Identification and Quantification of Microplastics Using Nile Red Staining. *Mar. Pollut. Bull.* **2016**, *113* (1–2), 469–476. https://doi.org/10.1016/j.marpolbul.2016.10.049.

(18) Erni-Cassola, G.; Gibson, M. I.; Thompson, R. C.; Christie-Oleza, J. A. Lost, but Found with Nile Red: A Novel Method for Detecting and Quantifying Small Microplastics (1 Mm to 20 Mm) in Environmental Samples. *Environ. Sci. Technol.* **2017**, *51* (23), 13641–13648. https://doi.org/10.1021/acs.est.7b04512.

(19) Nel, H.; Chetwynd, A. J.; Kelleher, L.; Lynch, I.; Mansfield, I.; Margenat, H.; Onoja, S.; Oppenheimer, P.; Sambrook Smith, G. H.; Krause, S. Detection Limits Are Central to Improve Reporting Standards When Using Nile Red for Microplastic Quantification. *Chemosphere* **2021**, *263*. https://doi.org/10.1016/j.chemosphere.2020.127953.

(20) Hidalgo-Ruz, V.; Gutow, L.; Thompson, R. C.; Thiel, M. Microplastics in the Marine Environment: A Review of the Methods Used for Identification and Quantification. *Environ. Sci. Technol.* **2012**, *46* (6), 3060–3075. https://doi.org/10.1021/es2031505.

(21) Trischler, H. The Anthropocene: A Challenge for the History of Science, Technology, and the Environment. *NTM Int. J. Hist. Ethics Nat. Sci. Technol. Med.* **2016**, *24* (3), 309–335. https://doi.org/10.1007/s00048-016-0146-3.

(22) Waters, C. N.; Zalasiewicz, J.; Summerhayes, C.; Fairchild, I. J.; Rose, N. L.; Loader, N. J.; Shotyk, W.; Cearreta, A.; Head, M. J.; Syvitski, J. P. M.; Williams, M.; Wagreich, M.; Barnosky, A. D.; An, Z.; Leinfelder, R.; Jeandel, C.; Gałuszka, A.; Ivar do Sul, J. A.; Gradstein, F.; Steffen, W.; McNeill, J. R.; Wing, S.; Poirier, C.; Edgeworth, M. Global Boundary Stratotype Section and Point (GSSP) for the Anthropocene Series: Where and How to Look for Potential Candidates. *Earth-Science Rev.* **2018**, *178* (August 2017), 379–429. https://doi.org/10.1016/j.earscirev.2017.12.016.

(23) Head, M. J. Formal Subdivision of the Quaternary System/Period: Present Status and Future Directions. *Quat. Int.* **2019**, *500* (May), 32–51. https://doi.org/10.1016/j.quaint.2019.05.018.

(24) Gałuszka, A.; Migaszewski, Z. M.; Rose, N. L. A Consideration of Polychlorinated Biphenyls as a Chemostratigraphic Marker of the Anthropocene. *Anthr. Rev.* **2020**, *7* (2), 138–158. https://doi.org/10.1177/2053019620916488.

(25) Jin, Y.-B.; Zhou, X.-D.; Xie, Y.-C.; Yu, Z.-G. Reconstruction of Temporal and Spatial Trends of Atmospheric Pollution Based on Polychlorinated Biphenyls Concentration Changes in Ombrotrophic Bogs. *J. Appl. Ecol.* **2021**, *32* (1), 309–316. https://doi.org/10.13287/j.1001-9332.202101.035.

(26) Capozzi, F.; Carotenuto, R.; Giordano, S.; Spagnuolo, V. Evidence on the Effectiveness of Mosses for Biomonitoring of Microplastics in Fresh Water Environment. *Chemosphere* **2018**, *205*, 1–7. https://doi.org/10.1016/j.chemosphere.2018.04.074.

(27) Roblin, B.; Aherne, J. Moss as a Biomonitor for the Atmospheric Deposition of Anthropogenic Microfibres. *Sci. Total Environ.* **2020**, *715*, 136973. https://doi.org/10.1016/j.scitotenv.2020.136973.

(28) Bancone, C. E. P.; Turner, S. D.; Ivar do Sul, J. A.; Rose, N. L. The Paleoecology of Microplastic Contamination. *Front. Environ. Sci.* **2020**, *8* (September), 1–20. https://doi.org/10.3389/fenvs.2020.574008.

(29) Rose, N. L.; Ruppel, M. Environmental Archives of Contaminant Particles. In *Environmental Contaminants: Using Natural Archives to Track Sources and Long-Term Trends of Pollution*; Blais, J. M., Rosen, M. R., Smol, J. P., Eds.; Springer: New York, 2015; pp 182–221. https://doi.org/10.1007/978-94-017-9541-8.

(30) Vasil'chuk, A. K. Taphonomic Features of Arctic Pollen. *Izv. Akad. Nauk Ser. Biol.* **2005**, *32* (2), 240–252.

(31) Dong, M.; Luo, Z.; Jiang, Q.; Xing, X.; Zhang, Q.; Sun, Y. The Rapid Increases in Microplastics in Urban Lake Sediments. *Sci. Rep.* **2020**, *10* (1), 1–10. https://doi.org/10.1038/s41598-020-57933-8.

(32) Mao, R.; Song, J.; Yan, P.; Ouyang, Z.; Wu, R.; Liu, S.; Guo, X. Horizontal and Vertical Distribution of Microplastics in the Wuliangsuhai Lake Sediment, Northern China. *Sci. Total Environ.* **2021**, *754*, 142426. https://doi.org/10.1016/j.scitotenv.2020.142426.

(33) Corcoran, P. L.; Norris, T.; Ceccanese, T.; Walzak, M. J.; Helm, P. A.; Marvin, C. H. Hidden Plastics of Lake Ontario, Canada and Their Potential Preservation in the Sediment Record. *Environ. Pollut.* **2015**, *204*, 17–25. https://doi.org/10.1016/j.envpol.2015.04.009.

(34) Lin, J.; Xu, X. M.; Yue, B. Y.; Xu, X. P.; Liu, J. Z.; Zhu, Q.; Wang, J. H. Multidecadal Records of Microplastic Accumulation in the Coastal Sediments of the East China Sea. *Chemosphere* **2020**, No. xxxx, 128658. https://doi.org/10.1016/j.chemosphere.2020.128658.

(35) Di, M.; Wang, J. Microplastics in Surface Waters and Sediments of the Three Gorges Reservoir, China. *Sci. Total Environ.* **2018**, *616–617*, 1620–1627. https://doi.org/10.1016/j.scitotenv.2017.10.150.

(36) Vaughan, R.; Turner, S. D.; Rose, N. L. Microplastics in the Sediments of a UK Urban Lake. *Environ. Pollut.* **2017**, *229*, 10–18. https://doi.org/10.1016/j.envpol.2017.05.057.

(37) Felismino, M. E. L.; Helm, P. A.; Rochman, C. M. Microplastic and Other Anthropogenic Microparticles in Water and Sediments of Lake Simcoe. *J. Great Lakes Res.* **2021**, *47* (1), 180–189. https://doi.org/10.1016/j.jglr.2020.10.007.

(38) Velasco, A. de J. N.; Rard, L.; Blois, W.; Lebrun, D.; Lebrun, F.; Pothe, F.; Stoll, S. Microplastic and Fibre Contamination in a Remote Mountain Lake in Switzerland. *Water (Switzerland)* **2020**, *12* (9), 1–16. https://doi.org/10.3390/W12092410.

(39) Lusher, A. L.; Buenaventura, N. T.; Eidsvoll, D. P.; Thrane, J.-E.; Økelsrud, A.; Jartun, M.

Freshwater Microplastics in Norway: A First Look at Sediment, Biota and Historical Plankton Samples from Lake Mjøsa and Lake Femunden; 2018. Norwegian Institute for Water Research: Norway, 2018. http://hdl.handle.net/11250/2588713 (accessed 2021-10-18)

(40) Turner, S.; Horton, A. A.; Rose, N. L.; Hall, C. A Temporal Sediment Record of Microplastics in an Urban Lake, London, UK. *J. Paleolimnol.* **2019**, *61* (4), 449–462. https://doi.org/10.1007/s10933-019-00071-7.

(41) Fischer, E. K.; Paglialonga, L.; Czech, E.; Tamminga, M. Microplastic Pollution in Lakes and Lake Shoreline Sediments - A Case Study on Lake Bolsena and Lake Chiusi (Central Italy). *Environ. Pollut.* **2016**, *213*, 648–657. https://doi.org/10.1016/j.envpol.2016.03.012.

(42) Su, L.; Xue, Y.; Li, L.; Yang, D.; Kolandhasamy, P.; Li, D.; Shi, H. Microplastics in Taihu Lake, China. *Environ. Pollut.* **2016**, *216*, 711–719. https://doi.org/10.1016/j.envpol.2016.06.036.

(43) Yuan, W.; Liu, X.; Wang, W.; Di, M.; Wang, J. Microplastic Abundance, Distribution and Composition in Water, Sediments, and Wild Fish from Poyang Lake, China. *Ecotoxicol. Environ. Saf.* **2019**, *170* (December 2018), 180–187. https://doi.org/10.1016/j.ecoenv.2018.11.126.

(44) Qin, Y.; Wang, Z.; Li, W.; Chang, X.; Yang, J.; Yang, F. Microplastics in the Sediment of Lake Ulansuhai of Yellow River Basin, China. *Water Environ. Res.* **2020**, *92* (6), 829–839. https://doi.org/10.1002/wer.1275.

(45) Zobkov, M.; Belkina, N.; Kovalevski, V.; Zobkova, M.; Efremova, T.; Galakhina, N. Microplastic Abundance and Accumulation Behavior in Lake Onego Sediments: A Journey from the River Mouth to Pelagic Waters of the Large Boreal Lake. *J. Environ. Chem. Eng.* **2020**, *8* (5), 104367. https://doi.org/10.1016/j.jece.2020.104367.

(46) Irfan, T.; Khalid, S.; Taneez, M.; Hashmi, M. Z. Plastic Driven Pollution in Pakistan: The First Evidence of Environmental Exposure to Microplastic in Sediments and Water of Rawal Lake. *Environ. Sci. Pollut. Res.* **2020**, *27* (13), 15083–15092. https://doi.org/10.1007/s11356-020-07833-1.

(47) Oni, B. A.; Ayeni, A. O.; Agboola, O.; Oguntade, T.; Obanla, O. Comparing Microplastics Contaminants in (Dry and Raining) Seasons for Ox- Bow Lake in Yenagoa, Nigeria. *Ecotoxicol. Environ. Saf.* **2020**, *198* (January), 110656. https://doi.org/10.1016/j.ecoenv.2020.110656.

(48) Clayer, F.; Jartun, M.; Buenaventura, N. T.; Guerrero, J. L.; Lusher, A. Bypass of Booming Inputs of Urban and Sludge-Derived Microplastics in a Large Nordic Lake. *Environ. Sci. Technol.* **2021**, *55* (12), 7949–7958. https://doi.org/10.1021/acs.est.0c08443.

(49) Bergmann, M.; Wirzberger, V.; Krumpen, T.; Lorenz, C.; Primpke, S.; Tekman, M. B.; Gerdts, G. High Quantities of Microplastic in Arctic Deep-Sea Sediments from the HAUSGARTEN Observatory. *Environ. Sci. Technol.* **2017**, *51* (19), 11000–11010. https://doi.org/10.1021/acs.est.7b03331.

(50) Sruthy, S.; Ramasamy, E. V. Microplastic Pollution in Vembanad Lake, Kerala, India: The First Report of Microplastics in Lake and Estuarine Sediments in India. *Environ. Pollut.* **2017**, *222*, 315–322. https://doi.org/10.1016/j.envpol.2016.12.038.

(51) Zbyszewski, M.; Corcoran, P. L. Distribution and Degradation of Fresh Water Plastic Particles along the Beaches of Lake Huron, Canada. *Water. Air. Soil Pollut.* **2011**, *220* (1–4), 365–372. https://doi.org/10.1007/s11270-011-0760-6.

(52) Boucher, J.; Faure, F.; Pompini, O.; Plummer, Z.; Wieser, O.; Felippe de Alencastro, L. (Micro) Plastic Fluxes and Stocks in Lake Geneva Basin. *TrAC - Trends Anal. Chem.* **2019**, *112*, 66–74.

https://doi.org/10.1016/j.trac.2018.11.037.

(53) Imhof, H. K.; Ivleva, N. P.; Schmid, J.; Niessner, R.; Laforsch, C. Contamination of Beach Sediments of a Subalpine Lake with Microplastic Particles. *Curr. Biol.* **2013**, *23* (19), 867–868. https://doi.org/10.1016/j.cub.2013.09.001.

(54) Zhang, K.; Su, J.; Xiong, X.; Wu, X.; Wu, C.; Liu, J. Microplastic Pollution of Lakeshore Sediments from Remote Lakes in Tibet Plateau, China. *Environ. Pollut.* **2016**, *219*, 450–455. https://doi.org/10.1016/j.envpol.2016.05.048.

(55) Maes, T.; Van der Meulen, M. D.; Devriese, L. I.; Leslie, H. A.; Huvet, A.; Frère, L.; Robbens, J.; Vethaak, A. D. Microplastics Baseline Surveys at the Water Surface and in Sediments of the North-East Atlantic. *Front. Mar. Sci.* **2017**, *4* (MAY), 1–13. https://doi.org/10.3389/fmars.2017.00135.

(56) Faure, F.; Demars, C.; Wieser, O.; Kunz, M.; De Alencastro, L. F. Plastic Pollution in Swiss Surface Waters: Nature and Concentrations, Interaction with Pollutants. *Environ. Chem.* **2015**, *12* (5), 582–591. https://doi.org/10.1071/EN14218.

(57) Blettler, M. C. M.; Ulla, M. A.; Rabuffetti, A. P.; Garello, N. Plastic Pollution in Freshwater Ecosystems: Macro-, Meso-, and Microplastic Debris in a Floodplain Lake. *Environ. Monit. Assess.* **2017**, *189* (11), 1–14. https://doi.org/10.1007/s10661-017-6305-8.

(58) González-Pleiter, M.; Velázquez, D.; Edo, C.; Carretero, O.; Gago, J.; Barón-Sola, Á.; Hernández, L. E.; Yousef, I.; Quesada, A.; Leganés, F.; Rosal, R.; Fernández-Piñas, F. Fibers Spreading Worldwide: Microplastics and Other Anthropogenic Litter in an Arctic Freshwater Lake. *Sci. Total Environ.* **2020**, *722*, 137904. https://doi.org/10.1016/j.scitotenv.2020.137904.

(59) Xiong, X.; Zhang, K.; Chen, X.; Shi, H.; Luo, Z.; Wu, C. Sources and Distribution of Microplastics in China’s Largest Inland Lake – Qinghai Lake. *Environ. Pollut.* **2018**, *235*, 899–906. https://doi.org/10.1016/j.envpol.2017.12.081.

(60) Ravi, S.; Bharath, M.; Achyuthan, H. Quantification of Microplastic in Red Hills Lake of Chennai City , Tamil. *Environ. Sci. Pollut. Res.* **2020**.

(61) Appleby, P. G. Chronostratigraphic Techniques in Recent Sediments. In *Tracking Environmental Change Using Lake Sediments: Basin Analysis, Coring, and Chronological Techniques*; Last, W. M., Smol, J. P., Eds.; Kluwer Academic Publishers: Dordrecht, The Netherlands, 2001; pp 171–203. https://doi.org/10.1007/0-306-47669-X_9.

(62) Goodsite, M. E.; Rom, W.; Heinemeier, J.; Lange, T.; Ooi, S.; Appleby, P. G.; Shotyk, W.; van der Knapp, W. O.; Lohse, C.; Hansen, T. S. High-Resolution AMS 14C Dating of Post-Bomb Peat Archives of Atmospheric Pollutants. *Radiocarb. An Int. J. Cosmogenic Isot. Res.* **2001**, *43* (2), 495–1146. https://doi.org/10.1017/S0033822200041163.

(63) Spalding, K. L.; Buchholz, B. A.; Bergman, L.-E.; Druid, H.; Frisén, J. Age Written in Teeth by Nuclear Tests. *Nature* **2005**, *437*, 333–334. https://doi.org/10.1083/437333a.
